# Implementation of Infrared-Activated Negative Electron Transfer Dissociation (IR-NETD) Using Xenon on a Quadrupole-Orbitrap-Quadrupole Linear Ion Trap Mass Spectrometer

**DOI:** 10.1021/jasms.5c00345

**Published:** 2025-12-15

**Authors:** Daniel J. Nesbitt, Keaton L. Mertz, Mitchell D. Probasco, Trenton M. Peters-Clarke, Trent J. Oman, John E. P. Syka, Scott T. Quarmby, Joshua J. Coon

**Affiliations:** † Department of Chemistry, 5228 University of Wisconsin-Madison, Madison, Wisconsin 53706, United States; ‡ Morgridge Institute for Research, Madison, Wisconsin 53715, United States; § Department of Biomolecular Chemistry, 5228 University of Wisconsin-Madison, Madison, Wisconsin 53706, United States; ∥ Eli Lilly and Co., Indianapolis, Indiana 46285, United States; ⊥ Thermo Fisher Scientific, San Jose, California 95134, United States; # National Center for Quantitative Biology of Complex Systems, Madison, Wisconsin 53706, United States

**Keywords:** photoactivation, dissociation, IR, laser, xenon, NETD, IR-NETD, RNA, complex

## Abstract

For tandem mass spectrometry, photoactivation capabilities enable a host of useful dissociation strategies. Herein we present the first implementation of an IR laser system on a next generation, quadrupole-Orbitrap-quadrupole linear ion trap hybrid MS system (Thermo Scientific Orbitrap Ascend). In addition, we establish xenon as an efficient source of radical cations for negative electron transfer dissociation (NETD) reactions. First, we take advantage of the instrument’s linear architecture to include a home-built photon detector to improve ease of use and laser alignment, along with straightforward introduction of xenon into the instrument. Second, we assess the instrument performance through infrared-activated NETD (IR-NETD) of a simple, unmodified 6-mer RNA molecule. Finally, we assessed the performance of IR-NETD for characterizing a synthetically complex small interfering RNA molecule, evaluating the effects of parameters including precursor charge state and IR laser power, demonstrating the benefits of concurrent IR photoactivation during NETD reactions. This straightforward instrumental approach represents a powerful and versatile tool for the characterization of complex biopharmaceutical molecules.

## Graphical abstract

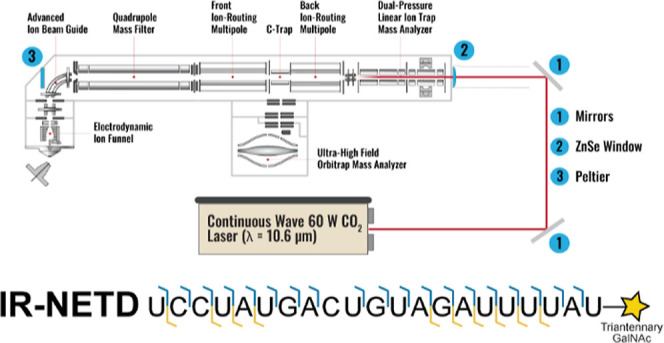

## Introduction

In recent years, photoactivation methods have become a valuable tool in tandem mass spectrometry,^1,2^ with applications ranging from small molecules^3–6^ to peptides^7–15^ to intact proteins.^16–23^ While ultraviolet photodissociation (UVPD) has been well developed, particularly for peptides and proteins, a number of promising applications of infrared multiphoton dissociation (IRMPD) have emerged more recently, including IRMPD for the characterization of native membrane proteins^18–20,24^ and multiplexed bottom-up proteomics.^25,26^ One additional benefit of an IR laser system is that the IR laser can be utilized to provide supplemental activation energy during ion–ion fragmentation events to improve the fragmentation efficiency of these ion–ion reactions.^27,28^

Electron transfer dissociation (ETD), the reaction of a reagent radical anion with cations, has become a fundamental fragmentation technique, particularly for peptides and proteins.^29,30^ While very effective, ETD does have drawbacks, mainly its charge-state dependence wherein precursors with low charge density undergo electron transfer without dissociation (ETnoD) due to noncovalent interactions.^31–35^ The addition of supplemental activation energy through either resonant (ETcaD) or beam-type (EThcD) collisional activation can lessen the degree of ETnoD and improve fragmentation efficiency; however, the resulting spectra become more complex, consisting of fragments generated through both collision- and electron-based dissociation pathways, as well as altered isotopic distributions due to hydrogen rearrangement.^36^ Previously, our lab has found that concurrent infrared photoactivation for the duration of the ETD reaction, termed activated-ion ETD (AI-ETD), is similarly effective at improving fragmentation efficiency compared to ETcaD and EThcD.^37–41^ The addition of IR photons increases the vibrational energy of the precursor ions, disrupting noncovalent interactions, resulting in improved dissociation efficiency when the ion–ion reaction occurs.^27,28,38^ Due to the recent widespread adoption of artificial intelligence (AI), we elect to rename this technique infrared activation (negative) electron transfer dissociation (i.e., IR-(N)­ETD). While IR-ETD is efficacious and well-studied for the analysis of cations, the counterpart of opposite polarity (negative ETD, NETD or IR-NETD), wherein a reagent radical cation reacts with an anion, has shown promise for the analysis of biomolecules with acidic moieties such as nucleic acids,^42,43^ oligosaccharides,^44^ and the acidic proteome.^45–47^

Most of our previous work involving NETD or IR-NETD has employed fluoranthene as the source of radical cations.^42–47^ Although both radical cations and anions can be generated from fluoranthene, the generation of radical cations is much less efficient than that of radical anions, resulting in decreased reagent brightness and longer reagent injection times for NETD compared to ETD. One other radical cation reagent source we have explored is sulfur pentafluoride. While it was effective for lower charge density precursors, it fouled the reagent ions source which resulted in rapid decrease in fluoranthene ETD reagent signal.^48^ Early investigations of NETD reagent sources found that xenon was a viable NETD reagent source, but fluoranthene was popularized due to its ability to perform both ETD and NETD.^49^ The key difference between these reagents is the ionization energy, which is directly proportional to the energy of the recombination event between the radical cation and precursor anion. For example, the ionization energies of fluoranthene and xenon are 7.9 and 12.1 eV, respectively.^48,50^ Thus, when reacting with the same precursor, the fluoranthene reaction will be less energetic than that of xenon—one study found that for phosphopeptide anions, xenon generates more neutral loss fragments, consistent with the more energetic NETD reaction of xenon.^50^ So, depending on the anion precursor of interest, one NETD reagent may be more suitable than another.

The consistent challenge in utilizing IR-NETD and other photoactivation methods is the implementation of the laser itself with evolving commercial instruments. Previous laser implementations have required modified gas pressures within the dual-pressure ion trap^11,28,51^ or the integration of a modified collision cell.^52,53^ While implementation on the first generation of quadrupole-Orbitrap-quadrupole linear ion trap (q-OT-QLT) hybrid mass spectrometer (Orbitrap Fusion Lumos)^54^ was somewhat straightforward,^38^ the highly linear architecture changes within the next generation q-OT-QLT hybrid mass spectrometer (Orbitrap Ascend)^55^ necessitated changes in laser implementation.

Herein we describe our strategy to attach an IR laser on the Orbitrap Ascend with modifications for improved ease of use. In addition, we detail implementation of xenon as a robust reagent for NETD fragmentation. Given the previously demonstrated efficacy of IR-NETD for the analysis of ribonucleic acids (RNA) with fluoranthene as the reagent cation source on an older laser implementation,^42,43^ we chose to evaluate the effectiveness of xenon for the characterization of RNA, with and without the use of supplemental IR activation, exploring both an unmodified 6-mer RNA and heavily modified 21-mer small interfering RNA (siRNA).

## Materials and Methods

### Mass Spectrometry and Instrument Modifications

A quadrupole-Orbitrap-quadrupole linear ion trap Tribrid MS system (Orbitrap Ascend, Thermo Fisher Scientific, San Jose, CA) was modified with a Synrad Firestar ti60 60 W CO_2_ continuous wave infrared laser (10.6 μm). To enable concurrent precursor activation with IR photoirradiation, laser firing was triggered through the charge state independent lens RF of an ETD scan. In addition, an IR thermopile detector was fabricated in house using a 0.05″ mild steel shim clamped to the ceramic substrate of a 15 × 15 mm single stage Peltier array module. The other side of the Peltier module was clamped to an aluminum heat sink that was in thermal contact with the chassis of the mass spectrometer to enable laser alignment (Figure 1). Additional details of the laser setup are discussed below (Figure 1). Furthermore, the instrument was modified to enable NETD using xenon as the reagent cation. A gas tank containing 1% Xe in helium (Toll Company, Plymouth, MN) was plumbed to the ETD reagent oven Beswick regulator to enable Xe radical cation generation by the internal ETD reagent ion source (Figure S2B). A modified version of MS Tune software (v. 4.2.4321) was used for additional calibrations to improve Xe reagent transmission. For all (IR-)­NETD scans, the maximum reagent injection time, reagent AGC target, and NETD reaction time were set manually through MS Tune. The reagent and analyte AGC targets were set to 1 × 10^5^ and 2.5 × 10^4^, respectively. All data were collected in profile mode at a resolving power of 120,000 (at 200 *m*/*z*). Precursors were isolated for tandem MS spectra with a 2.0 *m*/*z* isolation width, and all tandem MS spectra were the summed average of 25 transients unless otherwise noted.

**Figure 1:**
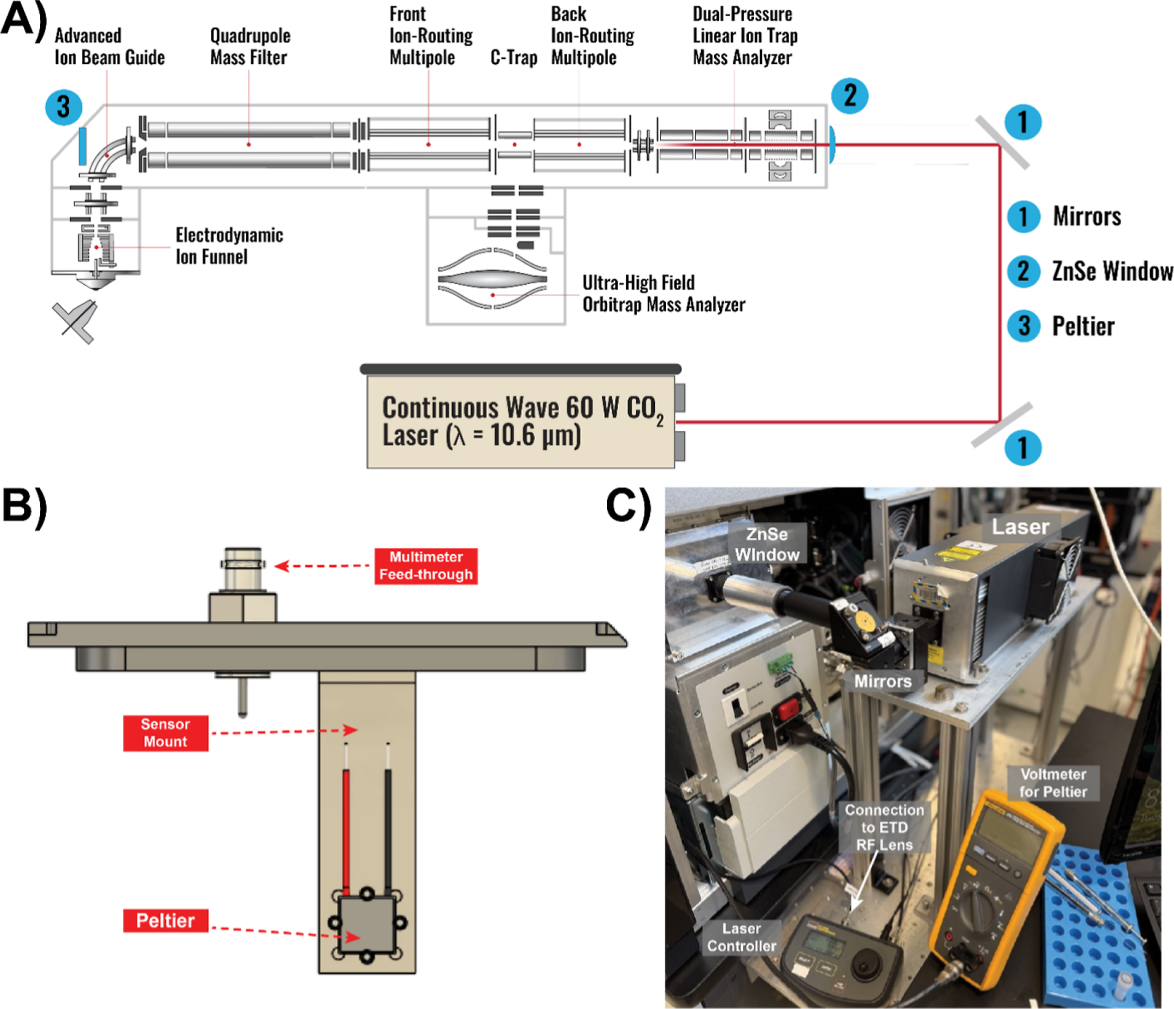
Modified Orbitrap Ascend Infrared Laser Setup. (A) Orbitrap Ascend instrument footprint depicting laser components and Peltier detector. (B) Schematic of Peltier for laser alignment. (C) Labeled image of laser setup at back end of instrument.

### Sample Preparation and Ionization

A 6-mer RNA standard (sequence: 5′-rGrUrArCrUrG-3′) was obtained from Integrated DNA Technologies (Coralville, IA), and the 21-mer siRNA containing a 3′-terminal triantennary *N*-Acetylgalactosamine (GalNAc) modification was obtained from Eli Lilly (Indianapolis, IN) (See Figure S1 21-mer sequence and structure). The RNA compounds were diluted to approximately 5 μM in 50:50 H_2_O/MeOH (LC–MS grade, Fisher Scientific) with 50 mM piperidine (Sigma-Aldrich) and infused into the mass spectrometer at 5 μL/min using a Chemyx Fusion 101 syringe pump. Electrospray ionization was achieved with a voltage of −3 kV relative to ground with the ion transfer tube held at 275 °C, and the ion funnel RF set to 60%. Source sweep gas was held at 4 arbitrary units to prevent Xe from reacting with oxygen (Figure S2C). The ionization energy of xenon (12.13 eV) is greater than that of molecular oxygen (12.07 eV), so the xenon radical cation will react with any molecular oxygen present, resulting in neutralization of the radical cation.^56^ Because the ionization energy of fluoranthene (7.9 eV) is less than that of molecular oxygen, fluoranthene suffers no oxygen reactivity issues.^56^ The sheath and auxiliary gas were adjusted manually to achieve stable spray.

### Data Analysis

All oligonucleotide sequencing spectra were analyzed using our in-house C# program, OligoSeq (https://github.com/coongroup/Transcriptomics).^42,43^ The user specifies the oligonucleotide sequence, as well as the position of any modifications within the sequence, to generate a list of theoretical fragments that is then matched against the experimental spectra. Fragments were annotated using nucleic acid dissociation nomenclature as defined by Mcluckey et al.^57^ (Figure 2A) and were matched with a mass tolerance of 10 ppm, with a signal-to-noise cutoff of 3. Further data analysis was conducted in MS Excel, and figures were generated using GraphPad Prism (v. 10.2.1) and Adobe Illustrator (v. 29.4). Sequence coverage denotes the percentage of phosphodiester bond cleavage using the eight sequence fragment types (a–d and w–z ions).

**Figure 2:**
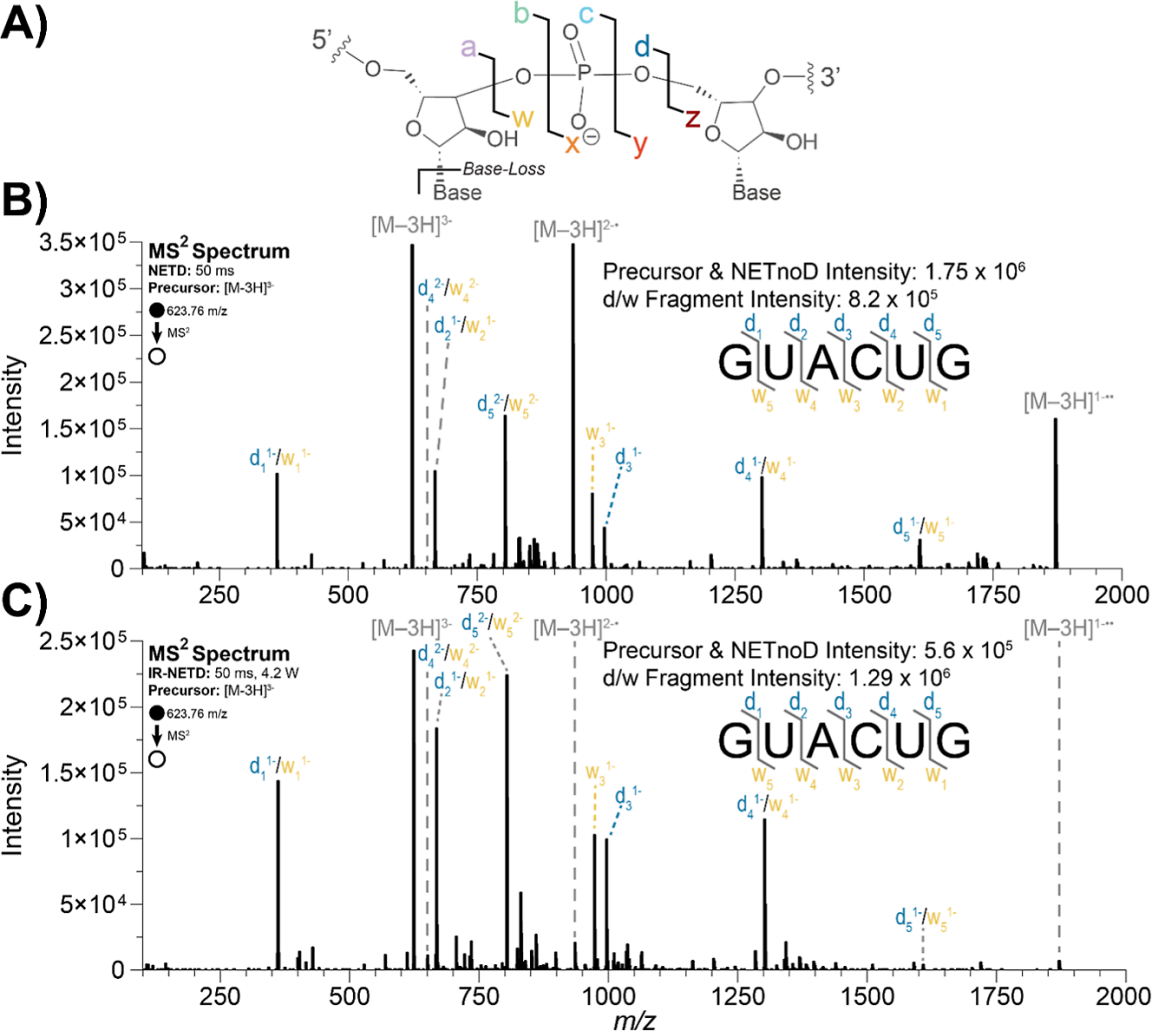
(A) Nomenclature for RNA backbone fragmentation as defined by Mcluckey et al.^57^ Annotated MS/MS spectra of an unmodified 6-mer RNA using (B) NETD and (C) IR-NETD.

## Results and Discussion

### Instrument Modifications

Building off our previous laser implementations, we sought to take advantage of the Orbitrap Ascend’s linear architecture to improve the robustness of alignment and ease of use of the laser system (Figure 1). The IR photon beam is guided by two mirrors to the ZnSe window at the rear of the linear ion trap (Figure 1A). In addition, we fabricated and attached a Peltier behind the bent flatapole in line with the beam path (Figure 1B). Once coarsely aligned, alignment was finely adjusted to maximize the voltage output from the Peltier. This ensured the IR beam was coaxially aligned with the ion optics. Enables straightforward laser alignment. To help maintain beam alignment, a metal base plate was attached directly to the mass spectrometer negating the drawbacks of a separate laser table (Figure 1C).^38^ In addition, the laser itself was secured and elevated to a height near the inlet of the linear ion trap to reduce the optical path length to decrease the difficulty of laser alignment. Overall, the secure metal base renders the setup both robust to incidental contact, and the Peltier renders laser realignment expedient and facile. To enable concurrent precursor activation with IR photoirradiation, the laser controller was connected to the charge state independent lens RF (Figure 1C) such that the laser fires for the full duration of the ion–ion reaction. The gas pressure throughout the linear ion trap was not changed from normal operating conditions. In addition, this setup enables standalone IRMPD by collecting an ETD/NETD scan and setting the reagent AGC and reagent maximum injection time to the minimum allowed values, and the ETD/NETD reaction time controls the IRMPD irradiation time. Furthermore, focusing lenses can be added within the optical path to improve IRMPD performance of precursors with lower IR-absorption cross sections.^17,38^

To implement xenon, a gas tank containing 1% Xe in helium was connected directly to the ETD reagent oven to generate the reagent radical cation, and a switch valve with the UHP N_2_ line was added to make it easy to switch back to fluoranthene (Figure S2A,B). The mass of the NETD reagent was changed to 131 *m*/*z* and additional NETD reagent transmission calibrations through the modified MS Tune software enabled consistent and bright reagent flux. The only significant limitation of xenon as the NETD reagent is that the radical cation can be quenched by reaction with oxygen—accordingly, all experiments require an enclosed source isolated from ambient atmosphere. In addition, we found that a low-level sweep gas setting of four arbitrary units was sufficient to prevent this depletion of the reagent radical cation (Figure S2C).

The combination of the robust IR laser setup and xenon NETD reagent enables myriad hybrid activation strategies for improved characterization of a variety of biomolecules under negative polarity. We sought to explore the capabilities of these instrument modifications for the characterization of RNA.

### NETD and IR-NETD with Xenon of a Short, Unmodified RNA Anion

Current generation siRNA therapeutics contain a host of synthetic modifications, including ribose, phosphate, and terminal modifications, all of which are designed to ameliorate drug performance characteristics such as in vivo stability or targeted drug delivery.^58–60^ While resonance type collisional activation is effective for sequence fragmentation of unmodified RNA, primarily through the generation of c and y fragments, it is rendered ineffective by 2′-ribose modifications.^61–63^ For modified RNA, beam-type collisional dissociation (higher-energy collisional dissociation, HCD) is required, but the spectra are complex as multiple collisional dissociation pathways result in c/y ion pairs and a-Base-Loss/w ion pairs.^61–63^ Electron-based fragmentation methods, such as NETD and IR-NETD, are effective for both modified and unmodified RNA, mainly generating d- and w-type sequence fragments.^59,60,64^

To examine the performance of NETD and IR-NETD with this new instrument configuration, we first characterized a simple, unmodified six-mer RNA (Figure S1A) to confirm instrument performance. Annotated NETD and IR-NETD tandem MS spectra of the triply charged precursor are shown in Figure 2B,C, respectively. Consistent with our previous laser setup using fluoranthene as the NETD reagent,^42^ both fragmentation methods achieved full sequence coverage, mainly generating d- and w-type fragment ions, though comparison between xenon and fluoranthene is difficult due to differences in instrumentation.

For NETD (Figure 2B), as expected, the NETnoD peaks were noticeably more intense than the d- and w-type sequence fragments (40% and 19% of the total ion current (%TIC), respectively). With concurrent infrared activation, however, the intensity of the NETnoD peaks in the IR-NETD spectrum (Figure 2C) was significantly reduced, accounting for just 16% TIC, or an approximate 3-fold decrease in relative intensity. Furthermore, the overall d- and w-type fragment intensity increased approximately 1.5-fold (37% of the TIC). The decrease in NETnoD peak intensity and increase in sequence fragment intensity improves overall spectral quality, clearly demonstrating the improved performance of IR-NETD relative to NETD.

### NETD and IR-NETD of a Heavily-Modified, Therapeutically Relevant siRNA Strand

Having demonstrated the efficacy of the instrument configuration, we sought to characterize a more complex, therapeutically relevant RNA molecule, a representative 21-mer siRNA sense strand containing various backbone and ribose modifications, also containing a 3′-terminal triantennary GalNAc targeting modification (Khvo sense strand) (Figure S1B). The triantennary GalNAc modification enables targeted drug delivery to liver hepatocytes through binding to the asialoglycoprotein receptor, resulting in drug internalization.^65,66^ As of January 2021 there were 31 different siRNA drugs containing triantennary GalNAc modifications across all three phases of FDA clinical trials.^65^

The Khvo sense strand was diluted to 5 μM in 50:50 H_2_O/MeOH with 50 mM piperidine to minimize salt adduct formation and to generate a wide range of charge states for investigation,^67^ ranging from *z* = −5 to *z* = −13, as shown in the full MS^1^ spectrum (Figure 3A). The fragmentation performance of NETD is highly dependent on precursor charge state,^31,32,35^ with improved performance at increasing charge states, so we first investigated the charge state dependence of NETD and IR-NETD. Annotated NETD and IR-NETD tandem MS spectra of the *z* = −6 precursor are shown in Figure 3B,C, respectively. Without supplemental IR activation, NETD failed to generate any sequence fragments, with the precursor and the NETnoD peak accounting for the bulk of signal observed at 19.67 and 52.92% TIC, respectively (Figure 3B).

**Figure 3:**
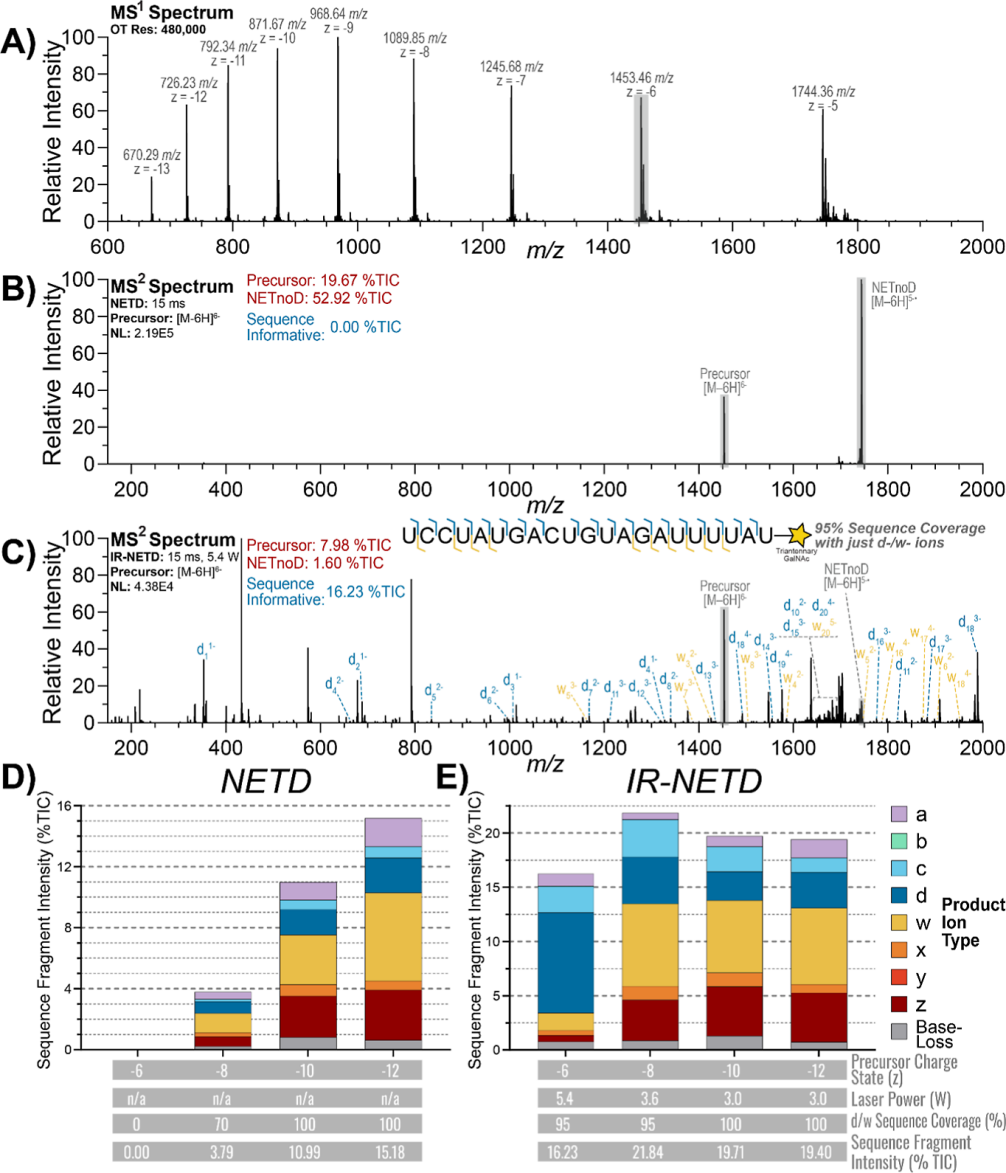
(A) Full MS^1^ spectrum of the Khvo Sense Strand. (B) NETD spectrum of the *z* = −6 precursor. (C) IR-NETD spectrum of the *z* = −6 precursor with d- and w-type sequence fragments and sequence coverage annotated. (D) Sequence fragment distribution, sequence coverage, and total sequence fragment intensity observed using NETD across multiple precursor charge states. (E) Sequence fragment distribution, sequence coverage, and total sequence fragment intensity observed using IR-NETD across multiple precursor charge states.

However, IR-NETD with 5.4 W of supplemental IR activation sufficiently disrupted the gas-phase noncovalent interactions of the precursor to generate consistent sequence fragmentation. Full sequence coverage was achieved, with sequence fragments accounting for 16.23% TIC, and the intensity of the precursor and NETnoD peak decreased to just 7.98 and 1.60% TIC, respectively (Figure 3C). In addition, 95% sequence coverage was achieved with just typical d/w fragments (Figure 3C).

For higher precursor charge states, the performance NETD without supplemental IR activation is improved (Figure 3D). For the *z* = −8 precursor NETD achieved 80% sequence coverage (compared to 0% for the z = −6) with sequence fragments accounting for 3.79% TIC. The higher charged *z* = −10 and −12 precursors both achieved complete sequence coverage, and sequence fragments accounted for 10.99 and 15.18% TIC, respectively, reflecting the improved fragmentation efficiency with NETD for more highly charged precursors. For the *z* = −10 and −12 precursors, w- and z-type ions accounted for most of the observed sequence fragment intensity, followed by a- and d-type ions. While d- and w-type ions are the typical sequence fragments for NETD of RNA, the presence of the phosphorothioate modifications at both ends of the Khvo sense strand promote the generation of a- and z-type ions as well.^43^

With the addition of supplemental IR activation, fragmentation performance improved across all precursor charge states (Figure 3E). Full sequence coverage was achieved for all precursor charge states tested, with sequence fragment intensity ranging from 16.23 to 21.84% TIC. For the *z* = −6 precursor, the 5′-containing sequence fragments (a/b/c/d) exhibited approximately 5-fold greater intensity than the 3′-containing sequence fragments (w/x/y/z). Conversely, for the *z* = −8, −10, and −12 precursors, the total intensity of 3′-containing sequence fragments was approximately twice that of 5′-containing fragments. For these higher charge state precursors, it is possible that the GalNAc modification could bear significant charge in the gas phase, altering the fragmentation behavior compared to lower precursor charge states in which most of the charge is concentrated throughout the RNA backbone. In addition, the optimal laser power for IR-NETD decreased with increasing precursor charge state, likely due to the improved NETD performance at higher precursor charge states, meaning less IR activation is required to improve the NETD reaction performance.

To further investigate the effect of laser power on fragmentation performance, we performed IR-NETD on the *z* = −10 precursor across a range of laser powers (Figure 4). As the laser power increased, the intensity of the precursor and NETnoD products, as expected, sharply decreased, however, at higher laser powers the relative intensity of sequence-informative fragments also decreased (Figure 4A–D). In addition, increasing laser power results in generally lower intensity peaks throughout the mass spectrum with several peaks making up most of the intensity within the spectrum (Figure 4D). Examining the distribution of identified sequence fragments (Figure 4E) reveals a noticeable shift in the fragment distribution when increasing the laser power during IR-NETD. Compared to NETD, an IR-NETD laser power of 3.0 W maintains complete sequence coverage but increases the identified sequence fragment intensity from 10.99 to 19.71% TIC. Additional laser power above 3.0 W resulted in a slight decrease in sequence coverage to 95%. At these laser powers, no sequence fragments were generated through cleavage of the last phosphodiester bond at the 3′ end of the molecule. At up to 4.8 W of laser power, the identified sequence fragment intensity remained close to 20% TIC, but there was a decrease to 15.38% TIC at a laser power of 5.4 W. At this laser power, dissociation through IRMPD begins to compete with the electron-based dissociation, resulting in an increase in the relative intensity of c, y, and base-loss fragments. Furthermore, the proportion of 3′-containing sequence fragments decreases, likely as a result of collision-based dissociation of the triantennary GalNAc modification. Ultimately, optimal laser powers for IR-NETD are below this threshold as the competition between electron- and collision-based dissociation results in overfragmentation and a decrease in both the intensity of identifiable sequence fragments and sequence coverage.

**Figure 4:**
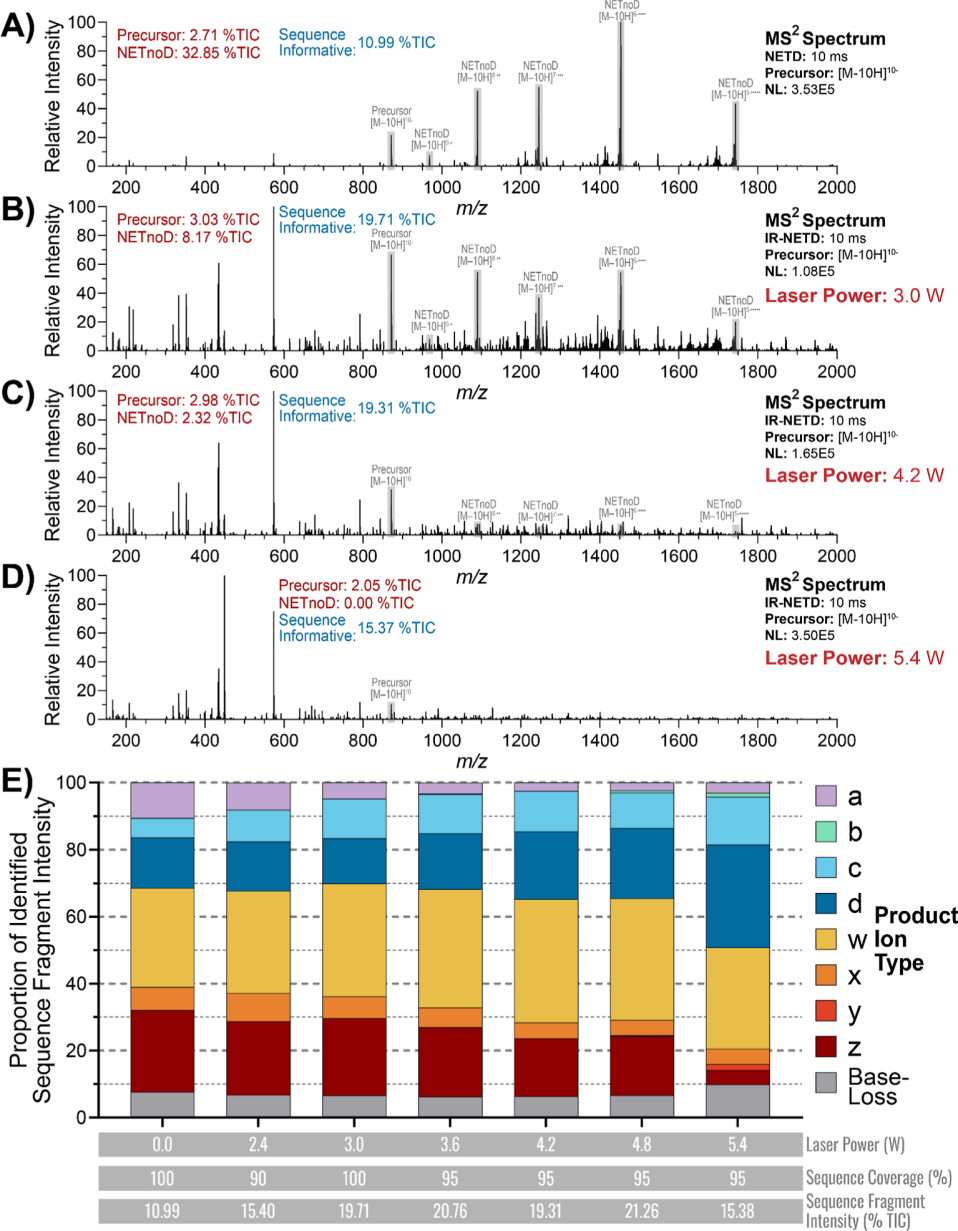
MS^2^ fragmentation spectra of the Khvo Sense Strand *z* = −10 precursor using (A) NETD and (B–D) IR-NETD at laser powers of (B) 3.0 W, (C) 4.2 W, and (D) 5.4 W. (E) Relative distribution of identified sequence fragments, sequence coverage, and total sequence fragment intensity of the *z* = −10 precursor with increasing laser power.

## Conclusions

Building on our previous IR laser implementations, we successfully affixed an IR laser to the newest generation of q-OT-QLT instruments, the more linear Orbitrap Ascend. We leveraged the linear architecture to add a Peltier at one end of the instrument to render laser alignment more facile and robust. Furthermore, we employed xenon as an alternative NETD reagent source. We have shown that xenon on its own is an effective NETD reagent for the analysis of RNA, with supplemental IR activation greatly improving fragmentation efficiency and sequence coverage. For more synthetically complex RNA molecules, NETD with xenon was effective on its own for highly charged precursors, and supplemental IR activation was able to recover fragmentation performance for less densely charged precursors. Lastly, we showed that the laser power can be adequately controlled to ensure minimal collisional activation of precursors.

These instrument modifications enable significant investigations into the characterization of complex RNA therapeutics. In addition, these modifications enable myriad activation methods such as CAD^62^ or IRMPD^68,69^ of the odd-electron NETnoD peaks generated from the xenon NETD reaction, investigations for which are underway. Additional investigations of how triantennary GalNAc modifications affect RNA backbone fragmentation are ongoing. Future work includes more targeted analysis of the fragmentation of different triantennary GalNAc modifications, aimed toward developing a broadly applicable LC–MS strategy for both backbone sequencing and terminal modification characterization utilizing IR-NETD and collision-based activation methods.

## Supplementary Material


