# Supplementary material for: Implementation of Infrared-Activated Negative Electron Transfer Dissociation (IR-NETD) Using Xenon on a Quadrupole-Orbitrap-Quadrupole Linear Ion Trap Mass Spectrometer
Source: J Am Soc Mass Spectrom. 2025 Dec 15;37(1):301–9. doi: 10.1021/jasms.5c00345 (PMC12784409; doi:10.1021/jasms.5c00345)
Supplement: Supplementary file 1 [file js5c00345_si_001.pdf]

**SUPPLEMENTAL MATERIAL**

# Implementation of Infrared-Activated Negative Electron Transfer Dissociation (IR-NETD) Using Xenon on a Quadrupole-Orbitrap-Quadrupole Linear Ion Trap Mass Spectrometer

Daniel J. Nesbitt,[1] Keaton L. Mertz,[1] Mitchell D. Probasco,[2,3] Trenton M. Peters-Clarke,[1] Trent J. Oman,[4] John E. P. Syka,[5] Scott T. Quarmby,[3,6] and Joshua J. Coon[1,2,3,6,*]

*To whom correspondence should be addressed: jcoon@chem.wisc.edu

1. Department of Chemistry, University of Wisconsin-Madison, Madison, WI 53706, United States
2. Morgridge Institute for Research, Madison, WI 53715, United States
3. Department of Biomolecular Chemistry, University of Wisconsin-Madison, Madison, WI 53706, United States
4. Eli Lilly and Co., Indianapolis, IN 46285, United States
5. Thermo Fisher Scientific, San Jose, CA 95134, United States
6. National Center for Quantitative Biology of Complex Systems, Madison, WI 53706, United States

## Supplemental Figures

**Figure S1.** Chemical structure of RNA molecules of study.

**Figure S2.** Setup for Xenon radical cation generation.

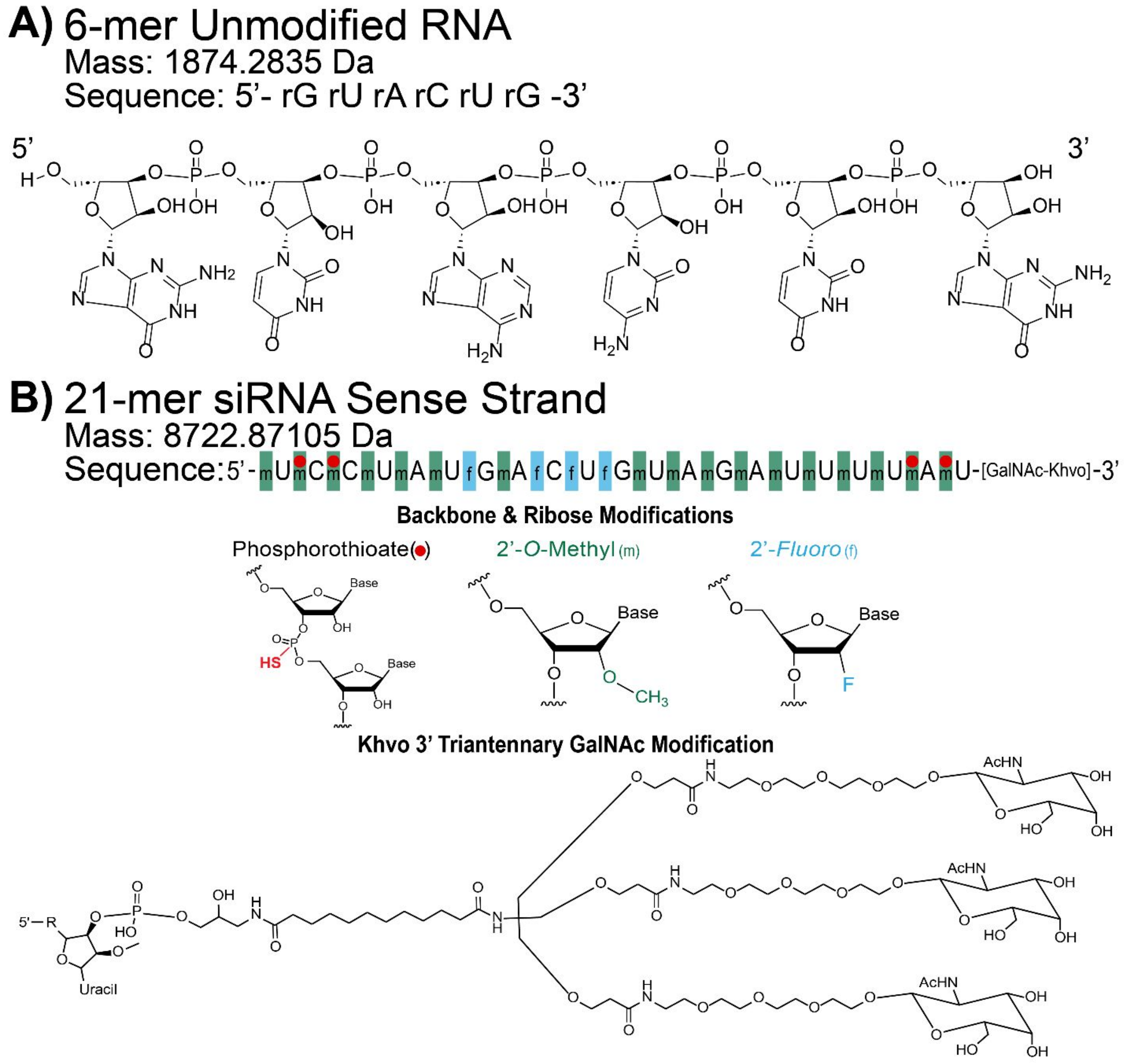


**Figure S1.** Structure of the RNA molecules of study. (A) Unmodified 6-mer RNA standard. (B) Representative 21-mer siRNA Sense Strand. Backbone and ribose modifications and sites are color coded, and the full structure of the tri-antennary GalNAc modification is depicted.

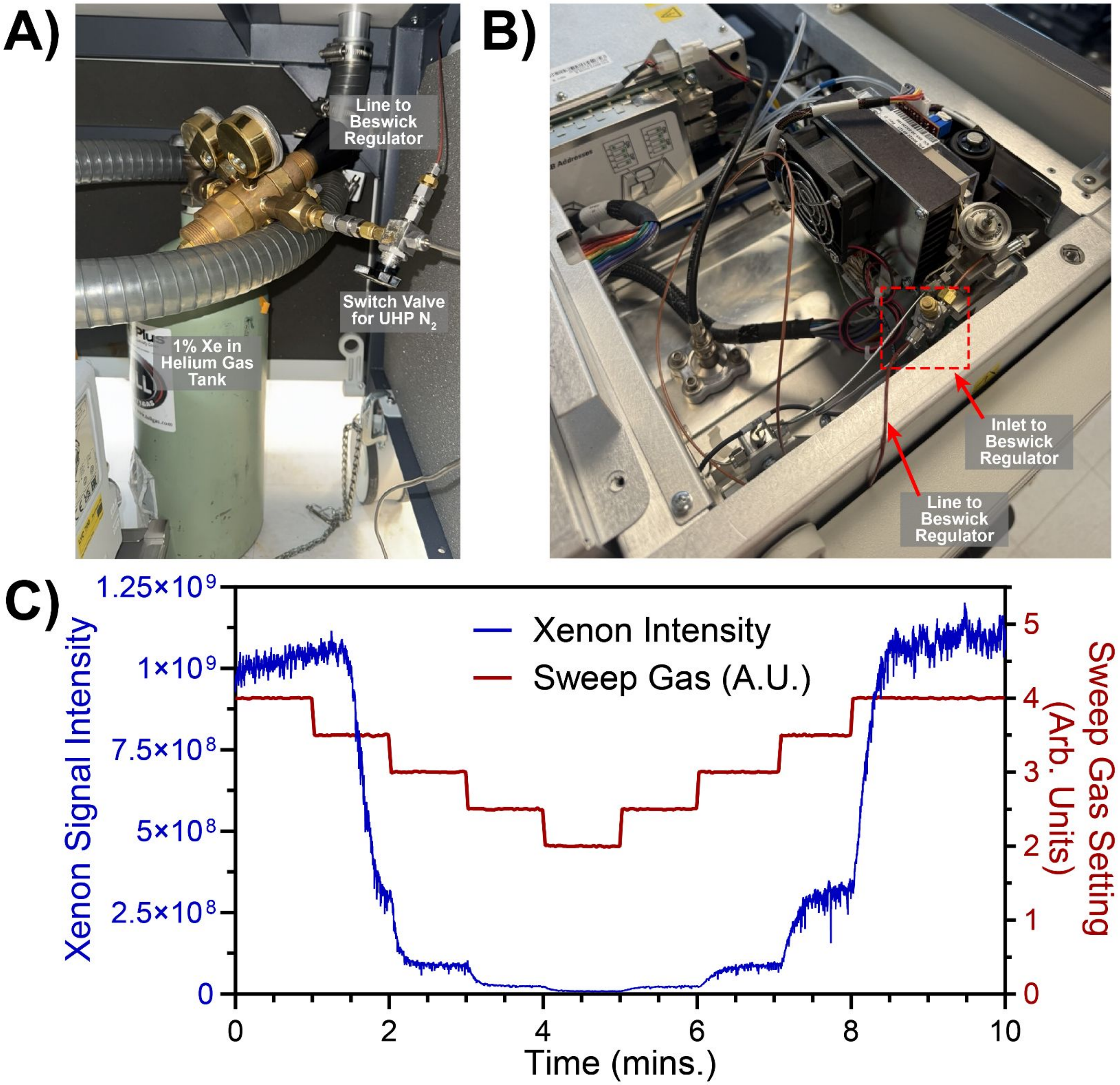


**Figure S2.** (A) Setup of the xenon gas tank and switch valve which allows operation with the standard nitrogen carrier gas for typical fluoranthene operation or with xenon. (B) Connection from the xenon gas tank to the ETD reagent oven Beswick Regulator. (C) Intensity of the most abundant isotope of the Xenon reagent cation vs Sweep Gas (arb. units), demonstrating the loss and recovery of the reagent intensity because of reactions with atmospheric oxygen.
